# Association between *HIF1A* rs11549465 polymorphism and risk of prostate cancer: a meta-analysis

**DOI:** 10.18632/oncotarget.16489

**Published:** 2017-03-22

**Authors:** Xiao-Dong Li, Hao Zi, Cheng Fang, Xian-Tao Zeng

**Affiliations:** ^1^ Department of Urology, Center for Evidence-Based Medicine, Management Office of Scientific Research and Postgraduate Affairs, Huaihe Hospital of Henan University, Kaifeng, China; ^2^ Center for Evidence-Based and Translational Medicine, Zhongnan Hospital of Wuhan University, Wuhan, China; ^3^ Department of Urology, Zhongnan Hospital of Wuhan University, Wuhan, China

**Keywords:** HIF1A, prostate cancer, polymorphism, risk, susceptibility

## Abstract

The hypoxia inducible factor 1-alpha (*HIF1A*) gene has been suggested to play a critical role in cancer progression, and the relationship between *HIF1A* rs11549465 polymorphism and risk of prostate cancer has been investigated in previous studies. Nevertheless, conflicting results have been obtained. Hence, we reevaluated this issue by means of this meta-analysis, with the purpose of providing more precise conclusion on this issue. The electronic databases of PubMed, EMBASE and Chinese National Knowledge Infrastructure (CNKI) as well as other sources were searched for relevant reports concerning on the role of *HIF1A* rs11549465 polymorphism in the occurrence of prostate cancer. The strength of the relationship was determined by calculating odds ratios (ORs) with corresponding 95% confidence intervals (95% CIs). Besides, subgroup analyses by ethnicity and source of control were further performed to examine this relationship. All statistical analyses were performed using STATA software 12.0. Although *HIF1A* rs11549465 polymorphism showed a tendency of increasing the risk of prostate cancer, no statistical significance was detected under any genetic models. Similar results were also revealed in subgroup analyses on the basis of ethnicity and control source. Our findings indicate that *HIF1A* rs11549465 polymorphism may not independently play a significant role in the occurrence of prostate cancer.

## INTRODUCTION

Prostate cancer is the second most common cancer in men all over the world and the fourth most frequent cancer overall, accounting for 11% of male cancers and 9% of cancer-related mortality [1–2]. The five-year survival rate is nearly 100% in patients with localized prostate cancer, but it's only 31% in those with distant metastases [3]. In the United States, prostate cancer led to 186,000 new cases and 28,600 deaths in 2008 [4]. The pathogenesis of prostate cancer is still not exactly known yet, but inheritance has been suggested to be the clearest one [5]. The risk of developing this cancer is two-fold higher among men having first-degree family members with prostate cancer than those without a family history of the cancer [6]. Some external factors, such as alcohol consumption, chronic inflammation, sexual behavior patterns, and exposure to ultraviolet light, have been put forward to be possibly correlated with the occurrence of the malignancy [7]. In addition, angiogenesis has also been demonstrated to play an important role in prostate carcinogenesis [8–9].

Angiogenesis, referring to the formation of new blood cells from pre-existing cells [10–11], plays an essential role in tumor development and metastasis, which has been indicated to exert negative effects on the disease status and prognosis of various tumors, including urological malignancies [12–13]. Several studies have shown that genetic polymorphisms in genes implicated in prostate angiogenesis may affect prostate cancer susceptibility [14–16]. The hypoxia inducible factor-1 (HIF-1) is composed of α and β subunits which serves to regulate the cellular response to hypoxia [17–18]. Besides, HIF-1 can also affect the expression of genes such as nitric oxide synthase 2 (NOS2), vascular endothelial growth factor, and erythropoietin (Epo) that are implicated in glucose metabolism, cell survival, iron metabolism, cell proliferation, cell survival, and angiogenesis [18–21]. Since the transcriptional activity of HIF-1 can be affected by the oxygen-regulated expression of HIF1A subunit [22], and the overexpression of HIF1A has been observed in many tumors, HIF1A has been suggested to impact the cancer onset [12, 18, 23]. A polymorphism rs11549465 in *HIF1A* gene has been proposed to be correlated with the occurrence risk of prostate cancer in several studies, but no definitive conclusion on the role of the single nucleotide polymorphism (SNP) in prostate cancer development has been drawn.

In the present study, we incorporated previously published studies to more systematically explore the impact of *HIF1A* rs11549465 polymorphism on prostate cancer risk.

## RESULTS

### Study characteristics

**Figure 1:**
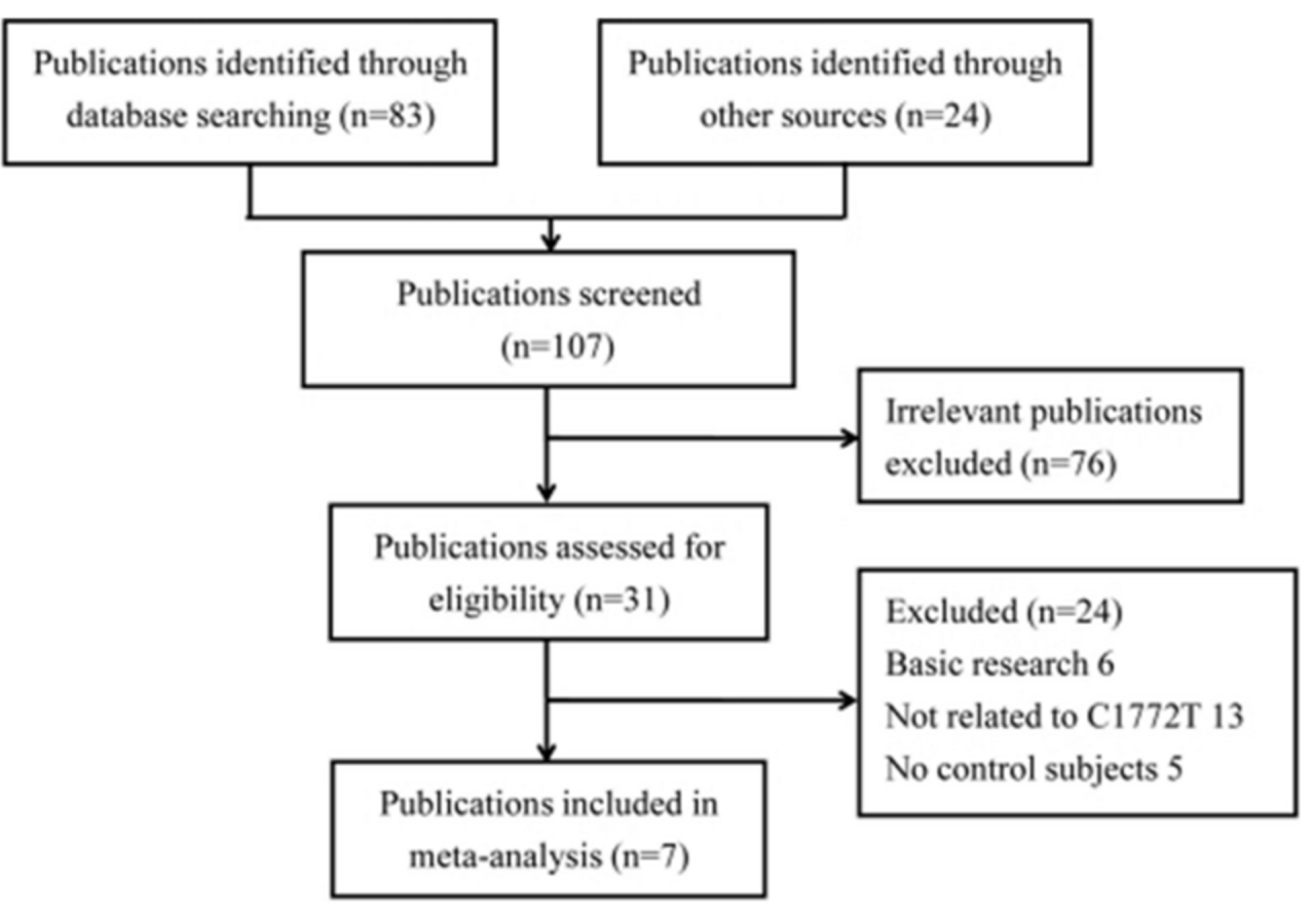
Flow diagram of selecting eligible studies for the meta-analysis

The selection process of eligible studies is presented in Figure 1. Initially, a total of 83 studies were obtained through publication search in electronic databases, and 24 articles were identified from other sources. However, 76 publications were deleted for obvious irrelevance. Among the remaining reports, 24 more were removed for basic research (*n* = 6), not involving *HIF1A* rs11549465 polymorphism (*n* = 13), and no controls (*n* = 5). As a consequence, a total of 4,570 cases and 4,820 controls were included in the present study [14, 24–29]. Table 1 describes principal characteristics of these included studies.

**Table 1:** Major characteristics of all studies included in the present study

First author, year	Country	Ethnicity	Control source	Sample size	Case					Control					HWE	Genotyping method
				Case/control	CC	CT	TT	C	T	CC	CT	TT	C	T		
Li, 2007	USA	Caucasian	PB	1041/1234	818	209	14	1845	237	995	221	18	2211	257	0.159	PCR-RFLP
Orr-Urtreger, 2007	Israel	Caucasian	PB	251/200	170	72	9	412	90	147	51	2	345	55	0.288	PCR-RFLP
Orr-Urtreger, 2007	Israel	Caucasian	PB	151/100	117	27	7	261	41	70	29	1	169	31	0.284	PCR-RFLP
Jacobs, 2008	USA	Caucasian	PB	1420/1450	1156	252	12	2564	276	1138	284	28	2560	340	0.041	MassARRAY
Foley, 2009	Ireland	Caucasian	PB	95/188	65	30	0	160	30	175	13	0	363	13	0.623	Sequencing
Li, 2012	China	Asian	HB	662/716	612	48	2	1272	52	659	57	0	1375	57	0.267	TaqManSNP
Fraga, 2014	Portugal	Caucasian	HB	754/736	579	164	11	1322	186	566	156	14	1288	184	0.399	TaqManSNP
Chau, 2005	USA	Mixed	PB	196/196	161	29	6	351	41	179	14	3	372	20	0.0002	Sequencing

PB, Population-based; HB, Hospital-based; HWE, Hardy-Weinberg Equilibrium; PCR-RFLP, Polymerase chain reaction-restriction fragment length polymorphism.

### Meta-analysis results

**Figure 2:**
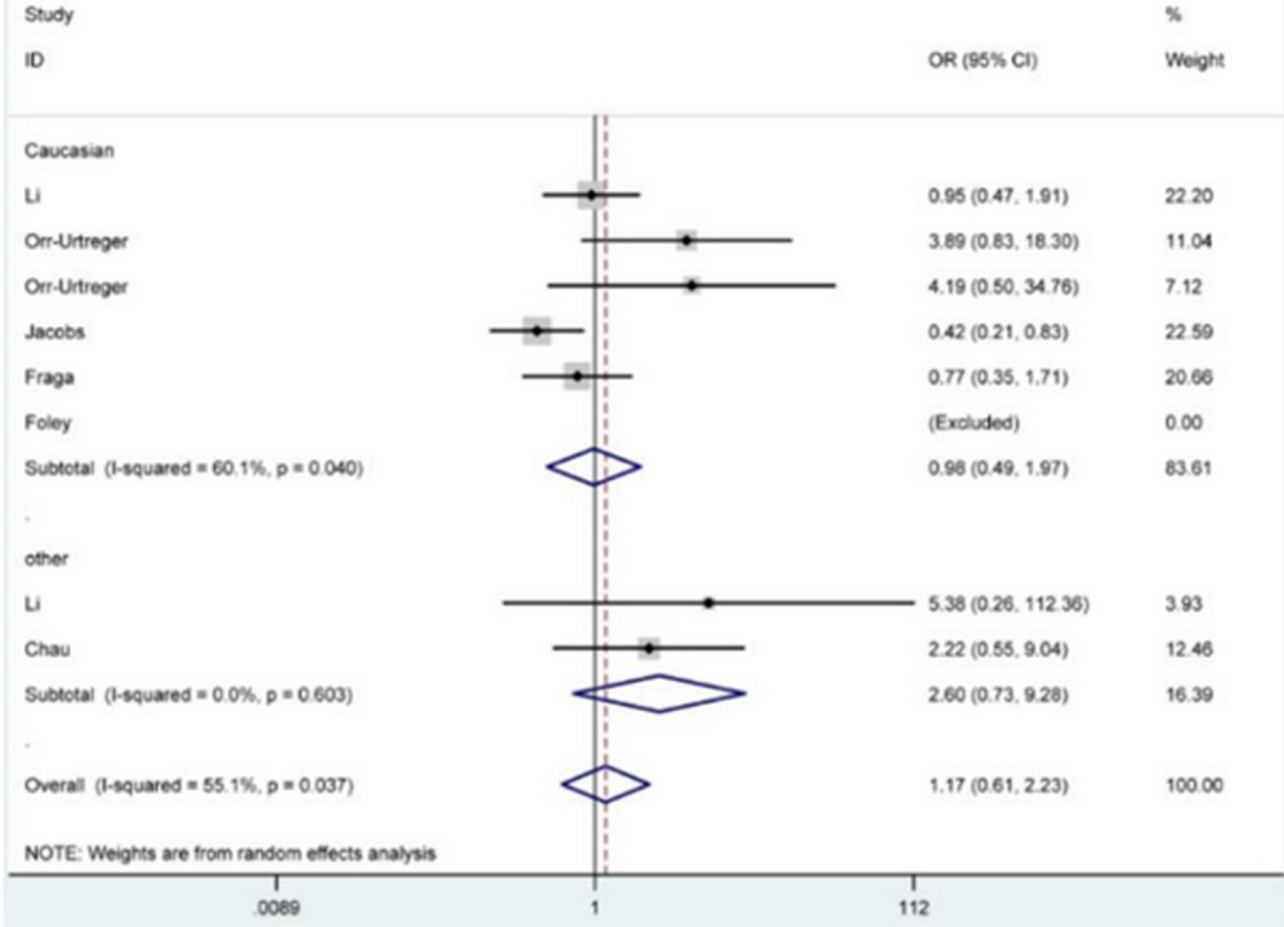
Forest plot for the correlation between HIF1A rs11549465 polymorphism and prostate cancer susceptibility under TT *versus* CC genetic model. The squares and horizontal lines correspond to the study-specific OR and 95% CI The area of the squares reflects the weight (inverse of the variance). The diamond represents the summary OR and 95% CI.

The relationship between *HIF1A* rs11549465 polymorphism and susceptibility to prostate cancer is illustrated in Table 2. Overall, in accordance with odds ratios (ORs) and 95% confidence intervals (95% CIs), no significant impact of the SNP on prostate cancer was detected under any of the five genetic contrasts TT *vs*. CC, TT+CT *vs*. CC, TT *vs*. CC+CT, T *vs*. C, and CT *vs*. CC [OR = 1.17, 95% CI = 0.61-2.23 (Figure 2); OR = 1.23, 95% CI = 0.93-1.64; OR = 1.15, 95% CI = 0.61-2.16; OR = 1.23, 95% CI = 0.95-1.60; OR = 1.21, 95% CI = 0.90-1.61]. A similar phenomenon was also revealed after subgroup analyses by ethnicity and control source.

**Table 2:** *HIF1A* rs11549465 polymorphism and risk of prostate cancer

Genetic contrast	Odds ratio (95% confidence interval) / *P* value for heterogeneity									
	Caucasian		Other-ethnicity		Population-based		Hospital-based		Total	
TT versus CC	0.98 (0.49, 1.97)	0.040	2.60 (0.73, 9.28)	0.603	1.30 (0.55, 3.07)	0.018	0.92 (0.44, 1.95)	0.221	1.17 (0.61, 2.23)	0.037
TT + CT versus CC	1.20 (0.86, 1.66)	0.000	1.42 (0.60, 3.38)	0.018	1.40 (0.93, 2.10)	0.000	0.99 (0.81, 1.21)	0.789	1.23 (0.93, 1.64)	0.000
TT versus CC+CT	0.98 (0.49, 1.96)	0.042	2.51 (0.72, 8.80)	0.563	1.28 (0.55, 2.96)	0.022	0.92 (0.44, 1.93)	0.218	1.15 (0.61, 2.16)	0.044
T versus C	1.19 (0.89, 1.59)	0.000	1.43 (0.66, 3.09)	0.022	1.39 (0.96, 2.01)	0.000	0.99 (0.82, 1.19)	0.995	1.23 (0.95, 1.60)	0.000
CT versus CC	1.18 (0.85, 1.64)	0.000	1.39 (0.56, 3.45)	0.019	1.35 (0.90, 2.05)	0.000	0.99 (0.80, 1.23)	0.602	1.21 (0.90, 1.61)	0.000

### Heterogeneity test

According to *P* values of the Chi-Square-based Q-statistical test, significant heterogeneity was observed in total analysis under all those genetic comparisons, so the random-effects model was adopted for OR evaluation. Since subgroup analyses by ethnicity and control source partially or totally eliminated the heterogeneity significance, we hypothesized that these two aspects might be able to explain a part of the sources of the heterogeneity.

### Sensitivity analysis

The sensitivity analysis was carried out through recalculating the pooled ORs after individual data-sets were removed one at a time, aiming to examine the influence of each single study on summarized results. Negligible changes in the final results confirmed the reliability of our results.

### Publication bias

**Figure 3:**
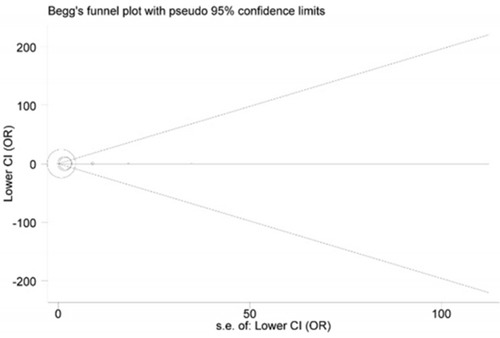
Begg's funnel plot of publication bias for HIF1A rs11549465 polymorphism Each point represents a separate study for the indicated association.

Publication bias across the selected studies was assessed with Begg's funnel plot and Egger's test. The funnel plots showed obvious symmetrical shapes (Figure 3), and results from Egger's test also supported the symmetry (*P* = 0.232), suggesting there was no significant publication bias.

## DISCUSSION

Prostate cancer is a rather common disease threatening the health of older males [30]. Global statistics show that, in 2008, the standardized morbidity and mortality rates of this cancer were 82.5/100,000 and 7.5/100,000, respectively [31–33]. The incidence of the disease was relatively low in China previously, but in recent years we have seen an increase in its incidence and mortality [30]. At present, no effective treatment method has been introduced for advanced-stage prostate cancer. Hence, it is urgent to identify both endogenous and exogenous factors contributing to invasion, proliferation, and migration of prostate cancer. According to relevant study results, some genetic variants may predispose people to prostate cancer [34–36]. The expression of HIF1A protein has been indicated to be positively correlated with the metastatic potential and cell growth rates [37–38], and its enhanced expression levels have been found in human high-grade prostate intraepithelial neoplasia (PIN) lesions as well as primary and metastatic prostate cancer [12, 39–40]. Several SNPs in the *HIF1A* gene have been identified, and the variant allele of the rs11549465 polymorphism (C-to-T substitution at locus +1772) has been shown higher transcriptional activity under both normoxic and hypoxic conditions when compared with the wild allele [41–42]. Therefore, many scholars have associated the SNP with the occurrence risk of prostate cancer. Nevertheless, the results from different researches remain inconsistent and even contradictory.

Chau et al. investigated the contribution of *HIF1A* rs11549465 polymorphism to the occurrence of androgen-independent prostate cancer (AIPC), and found an apparent difference in the genotype distribution between AIPC patients and control subjects, so they concluded that the SNP might be involved in the susceptibility to AIPC [24]. Similarly, in a study by Foley et al., the heterozygous genotype CT was identified to be a risk factor for clinically localized prostate cancer [25]. Besides, a replicated association was revealed as well among Jewish people in a research by Orr-Urtreger et al [28]. Nevertheless, Fraga et al. suggested that *HIF1A* rs11549465 polymorphism was not related to the risk of prostate cancer [29]. In addition, Jacobs et al. and Li et al also insisted that the SNP had no significant impact on susceptibility to prostate cancer in their studies [14, 26].

Above controversies over the role of *HIF1A* rs11549465 polymorphism in prostate cancer incidence may be due to several reasons. First of all, these studies described different types of prostate cancer. Secondly, the studies were carried out among people with different genetic backgrounds. Thirdly, different interfering environmental factors might be involved in the final results. Last but not least, studies with a small number of participants might get biased results.

In the present study, we found no statistically significant relationship between *HIF1A* rs11549465 polymorphism and the prostate cancer risk under any genetic comparisons, which was also true for subgroup analyses according to ethnicity and control source. Compared to the above-mentioned studies, the present meta-analysis had the advantage of larger sample size. However, some limitations in this study still should be addressed. To begin with, the influences of other relevant components such as age, gender, and smoking as well as their interactions with *HIF1A* rs11549465 polymorphism on prostate cancer occurrence were not analyzed due to the lack of original information. Second, meta-analysis is a secondary analysis and the heterogeneity is the major issue in genetic studies [43–44]. In our study, significant heterogeneity existed between the included studies, which might reflect differences in selection criteria, patients’ ethnicity, control source and analysis methodologies. Moreover, insufficient data provided in included studies restricted further evaluation of potential impacts of this polymorphism on aggressive prostate cancer and response to hormonal treatment, which might play an important role in the severity of the clinical disease. Next, only studies published in English or Chinese language were incorporated into the present study, so some potentially relevant data unpublished or published in other languages might be missed, thus leading to publication bias to some extent though it was not detected. Then, the majority of included studies offered information about Caucasian populations, which might cause selective bias. Therefore, the findings from this study need to be applied with prudence.

Taken together, the present meta-analysis manifests that *HIF1A* rs11549465 polymorphism may not be an independent risk factor for prostate cancer. However, due to the above limitations, our conclusions should be verified by multi-center studies with larger sample sizes based on multiple ethnic groups. Importantly, more attention should be paid to the roles of polymorphisms and their genetic variants in clinical aggressiveness and therapeutic response in future work.

## MATERIALS AND METHODS

This meta-analysis was reported according to the Preferred Reporting Items for Systematic Reviews and Meta-Analysis (PRISMA) statement [45] (PRISMA Checklist see Supplementary Table S1).

### Literature retrieval

The electronic databases of PubMed, EMBASE and Chinese National Knowledge Infrastructure (CNKI) as well as other sources were searched for relevant reports concerning on the role of *HIF1A* rs11549465 polymorphism in the occurrence of prostate cancer. The following search terms were adopted: “*HIF1A*” or “*HIF-1α*”, “prostate cancer” or “prostatic cancer”, and “polymorphism” or “variant” or “mutation” or “SNP”. Additionally, the references of all pertinent articles were manually checked for other relevant articles.

### Eligibility criteria

Eligible studies enrolled in the meta-analysis were required to fulfill the following criteria: (1) evaluating the association between *HIF1A* rs11549465 polymorphism and risk of prostate cancer; (2) with a case-control design; (3) published in English or Chinese language; and (4) containing sufficient data on genotype and/or allele frequencies both in case and control groups. Studies with any one of the following characteristics were excluded: (1) with only cases; (2) comment, review, or abstract; (3) animal study; and (4) offering duplicated content.

### Data extraction

The following items were extracted by two independent investigators using a specific sheet: name of the first author, publication year, country, ethnic line, control source, method for genotyping, total numbers of cases and controls, genotype and/or allele frequencies in cases and controls, and *P* values for Hardy-Weinberg Equilibrium (HWE) in control groups. Conflicting opinions over extracted data were resolved by discussion between the two investigators so as to reach a consensus.

### Statistical analysis

STATA software (version 12.0) was applied to perform all statistical calculations in this meta-analysis. *P* < 0.05 was considered as statistical significance. Whether genotype distribution in control group was in accordance with HWE was assessed by Chi-square test. ORs and 95% CIs were calculated to examine the strength of correlation between *HIF1A* rs11549465 polymorphism and prostate cancer susceptibility. The significance of pooled ORs was determined by Z test. Degree of heterogeneity across studies was evaluated by Chi-square-based Q test. In case of *P* < 0.05, indicating significant heterogeneity, random-effects model was applied to calculate pooled ORs; otherwise, fixed-effects model was used. The stability of final results was assessed by conducting sensitivity analysis. Begg's funnel plots and Egger's linear regression test were carried out to determine the underlying publication bias.

## SUPPLEMENTARY MATERIALS TABLE


